# A simple and efficient micrografting method for stably transformed *Nicotiana attenuata *plants to examine shoot-root signaling

**DOI:** 10.1186/1746-4811-7-34

**Published:** 2011-10-20

**Authors:** Variluska Fragoso, Hannah Goddard, Ian T Baldwin, Sang-Gyu Kim

**Affiliations:** 1 Department of Molecular Ecology, Max-Planck-Institute for Chemical Ecology, Hans-Knöll-Straße 8, D-07745 Jena, Germany; 2 MitoSciences, Inc., Eugene, Oregon 97403, USA

**Keywords:** Grafting, *Nicotiana attenuata*, root and shoot signaling, systemic signals

## Abstract

To adjust their development to the environment, plants rely on specific signals that travel from shoot to root and *vice versa*. Here we describe an efficient micrografting protocol for *Nicotiana attenuata*, a useful tool for identifying these signals and understanding their functions. Additionally we analyzed transcript accumulation profiles of scions and rootstocks of grafts performed with wild-type and stably transformed *N. attenuata*. Our results are consistent with the source-to-sink movement of an sRNA silencing signal.

## Background

Many studies have shown that plants use long-distance or systemic signals to coordinate and adjust their growth. These signals convey messages throughout the whole plant, from sensor to effector tissues or organs, and they seem to operate with great specificity which can depend not only on the message itself but also on the spatial and temporal scales over which they act [1]. One of the interesting examples of long-distance signaling in plants is activated by herbivore attack and results in the production of a complex bouquet of plant defenses. Plants are capable of priming defenses systemically in tissues that are distal to the sites of attack, suggesting that an herbivory alert signal is transmitted from attacked to unattacked leaves and roots [2].

Grafting has provided important insights into the study of these systemic wound signals in plants. Grafting a JA biosynthesis mutant with a JA response mutant clearly showed that the production of jasmonic acid (JA) in damaged leaves and the perception of JA by distal leaves are necessary for inducing systemic responses [3]. Moreover, the *de novo *biosynthesis of JA in systemic leaves was further determined not to be required for the systemic transmission of the wound signals [4]. This research focused on the long-distance communication within shoots, and there remains much to be learned about the role of roots in the production and propagation of these important signals throughout the plant that mediate ecological interactions [5].

A wild tobacco, *Nicotiana attenuata *has been studied in plant-herbivore interaction in its natural habitat, the Great Basin Desert of Utah. Several local and systemic defense responses are induced in *N. attenuata *during herbivore attack. Defense traits, which include trypsin proteinase inhibitors (TPI) and specific volatiles, such as trans-α-bergamotene produced by terpene synthases (TPS), are increased in *N. attenuata *when this plant is attacked by herbivores or elicited by herbivore-specific elicitors [6-8]. A notable example is of nicotine, which requires the activity of putrescine *N*-methyltransferase (PMT) in the roots for its synthesis and accumulates the alkaloid in leaves and other above-ground parts of tobacco plants [9]. This alkaloid restrains the consumption of *N. attenuata*'s leaves by its attackers, even by *Manduca sexta*, a specialist and nicotine-tolerant herbivore [10]. The identity of the signal which travels from attacked leaves and activates nicotine production in the roots, and whether this signal is the same that activates other systemic defenses in undamaged leaves are questions yet to be addressed [1].

Research in plant ecology has been greatly enhanced by the manipulation of gene expression in stably transformed plants. Isogenic lines expressing either low or high levels of transcripts for a particular gene provide a powerful tool to dissect its organism-level function and fitness consequences of the gene under real-world conditions [11]. However, one drawback of such genetic changes is when the transgene is driven by nonspecific promoters which therefore ectopically express transgenes or silencing constructs, preventing tissue- or organ-specific manipulations.

Here, we describe a simple and highly efficient micrografting protocol for *N. attenuata*, and characterize the influence of the grafting procedure on plant growth. Given that the graft junction is located between the shoots and roots of grafted plants, this technique allows for the independent manipulation of the below- and above-ground parts and the study of their interplay in *N. attenuata *for defense. Our results represent the first step towards the study of shoot-to-root interplay by micrografting in *N. attenuata*.

## Results

### Simple micrografting method for *N. attenuata *has a high success rate

**Figure 1:**
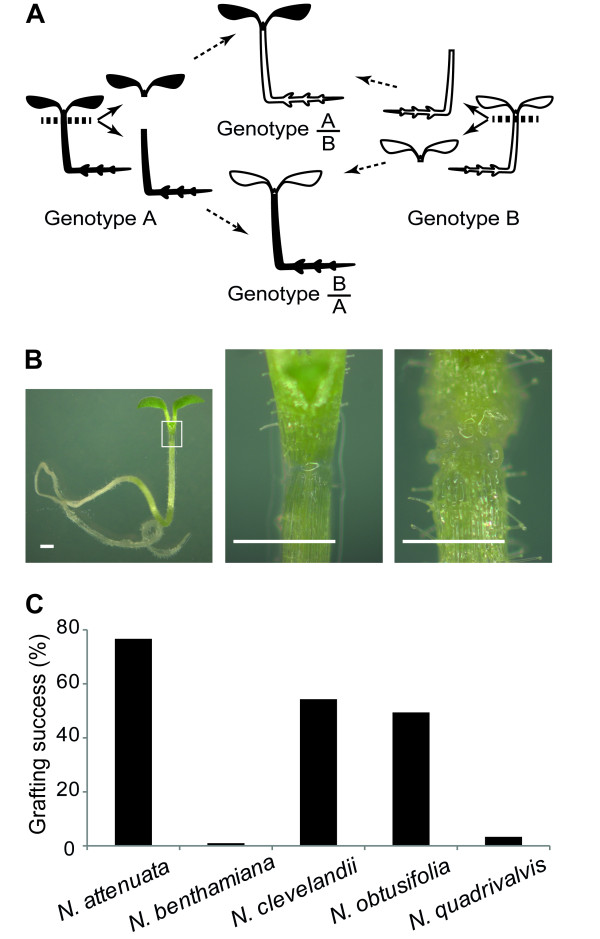
**Micrografting in *Nicotiana *spp**. (A) Schematic representation of micrografting. Graft A/B possesses scions from plant genotype A (black) fused to rootstocks from plant genotype B (white) and *vice versa *for graft B/A. (B) Pictures from right to left: one-week-old seedling of *N. attenuata *immediately after grafting; detail of junction portion of the hypocotyl marked in the black square on picture on the right; same seedling five days after grafting. Bars represent 500 μm. (C) Micrografting success rate for different *Nicotiana *species. *N. clevelandii*, n = 50; *N. attenuata, N. benthamiana, N. obtusifolia *and *N. quadrivalvis*, n = 100.

Preliminary cleft grafting experiments with adult plants of *N. attenuata *resulted in low success rate (approximately 10 ~ 15%). Therefore, we tested a method using plants in an earlier phase of development. One-week-old seedlings (approximately 2 mm in height) of different *Nicotiana *genotypes grown on agar plates were excised below the apical meristem (Figure 1A, B) to prevent adventitious rooting [12]. With a stereomicroscope and the seedlings lying horizontally on the media, the scion of one seedling was placed as close as possible to the rootstock of another. To stabilize the contact between scions and rootstocks, small blocks of agar were placed over the junction of the grafted seedlings. Five days after grafting, a visually apparent connection between the combined parts was observed, surrounded by feeble callus growth (Figure 1B).

Micrografting efficiency varied dramatically among different species of *Nicotiana *(Figure 1C). *N. attenuata *proved to be the most suitable among the species tested for this procedure, with an 80% success rate, as scored one week after grafting. The lowest grafting success rate was observed with *N. benthamiana *(ca. 1%) in which scions tended to produce roots under these *in vitro *grafting and growth conditions.

### Micrografting does not affect *N. attenuata *growth and development

**Figure 2:**
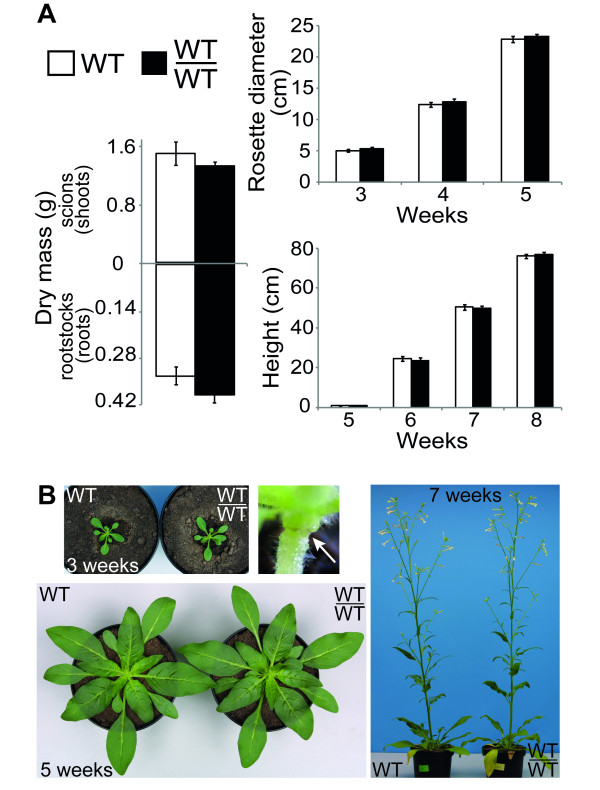
**Growth comparisons between intact WT and grafted WT/WT plants of *N. attenuata***. (A) Rosette diameter and height of WT and WT/WT plants and dry mass of shoots and roots of 6 week-old elongated WT and WT/WT plants did not differ significantly (Welch's *t*-test, n = 10). (B) Pictures of 3, 5 and 7-week-old WT (left) and WT/WT (right) plants. The top middle picture shows a detail of the grafted junction of a 3-week-old WT/WT plant (arrow).

Growth parameters of scions and rootstocks of grafted WT/WT *N. attenuata *plants (chimeras are named in a shoot/root manner throughout this paper) were compared weekly to intact WT individuals and no significant differences were observed (Figure 2). Rosette diameter and height (Figure 2A, B) as well as the length of the longest leaf (data not shown) of grafted WT/WT plants were of similar sizes compared to intact WT plants. The same was also true for biomass comparisons: shoot (Welch's *t*-test; *p *= 0.34) and root (Welch's *t*-test; *p *= 0.14) dry mass were similar in intact versus grafted plants (Figure 2A). Comparisons of the number of flowers (Welch's *t*-test; *p *= 0.94) (Figure 2B), capsules (Welch's *t*-test; *p *= 0.17) and seeds per capsule (Welch's *t*-test; *p *= 0.31) (data not shown) were also not significantly different.

The graft junction of three-week-old grafted plants was located just below the rosette (Figure 2B, top middle picture). This means that for this work, scions refer to whole shoot or above-ground portions of grafted plants, while rootstocks refer to the entire root system.

### Inverted repeat construct of scions affects the transcript accumulation in WT rootstocks

The main reason for establishing a grafting method for *N. attenuata *is to manipulate defense-related shoot-root signaling in lines already developed by our group (Additional file 1). Such stably transformed plants harboring constructs designed either to silence (antisense - *as *or inverted repeat - *ir*) or overexpress (*ov*) a particular gene are the main approaches by which gene function can be studied in *N. attenuata *system. However, the value of micrografting is mainly limited by the transmission of signals through the graft site [13]. Therefore, to validate the utility of the grafting method using these lines, we verified whether the transcript accumulation of WT scions and rootstocks was altered by their transgenic grafted counterpart. We made use of *TPI *and *PMT *genes as innate reporters of the up- and down-ward spread of silencing since the endogenous expression of these target genes in *N. attenuata *are particularly useful for this purpose: *TPI *is mainly expressed in shoots and *PMT *in roots [14]. We further investigated if the ectopically overexpressed (*ov*) mRNA of a foreign gene could also be transmitted to WT scions or rootstocks. Importantly, these lines are not morphologically distinguishable from WT *N. attenuata *plants [7,10,15], hence the effects of grafting on plant growth of these transgenic lines were comparable to those observed in grafts performed with WT plants (Figure 2A, B).

**Figure 3:**
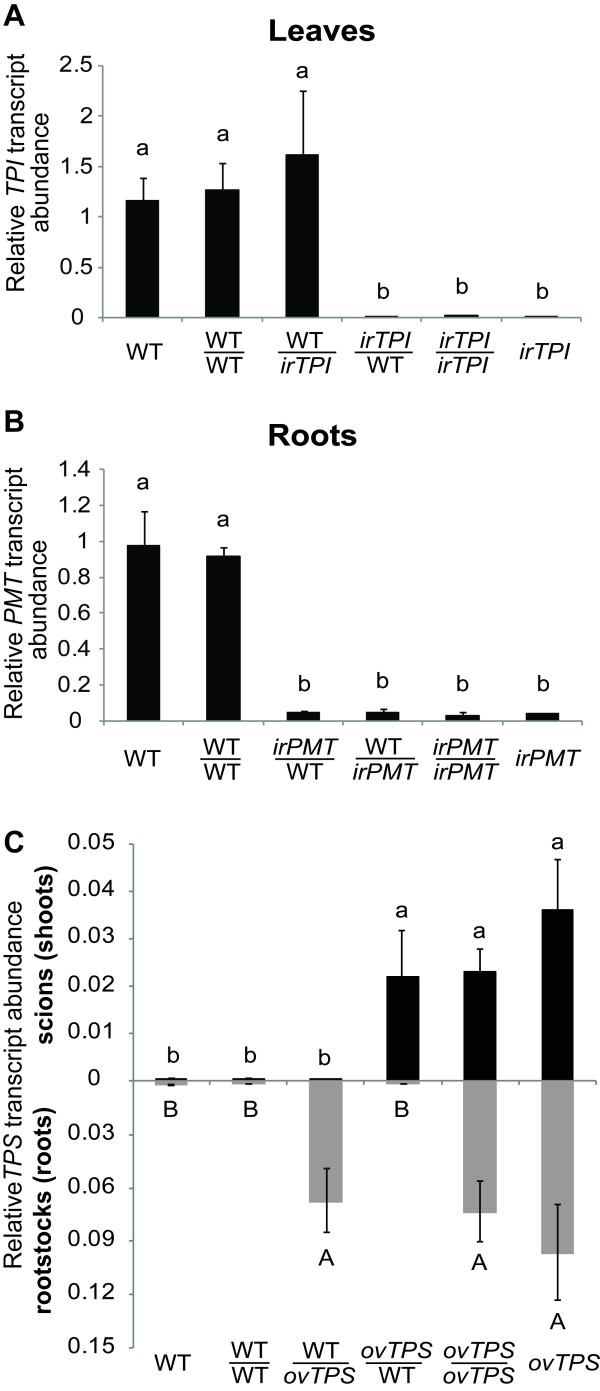
**Molecular analysis of leaves and roots of grafted plants transformed with different RNAi and overexpression constructs**. (A) *TPI *(normally expressed only in scions) transcript levels of leaves (scions) of intact or grafted WT and *irTPI*-silenced plants. (B) *PMT *(normally expressed only in roots) transcript levels of roots (rootstocks) of intact or grafted WT and *irPMT*-silenced plants. (C) *TPS *transcript levels of leaves (scions) and roots (rootstocks) of intact or grafted WT and *ovTPS*-overexpressing plants, in which a *TPS *from *Z. mays *was ectopically expressed behind a 35S promoter. Bars sharing same letters do not significantly differ (Kruskal-Wallis test followed by Fisher's PLSD, n = 5).

As expected [16], accumulation levels of *TPI *transcripts in leaves of *irTPI *plants (Figure 3A) were found to be dramatically reduced to below 1% of WT *TPI *transcript levels (Fisher's PLSD test; *p *< 0.0001). Likewise, *PMT *transcript levels in roots of *irPMT *plants (Figure 3B) were also found to be reduced to less than 5% of those found in the roots of WT plants (Fisher's PLSD test; *p *< 0.01), as previously characterized by Steppuhn et al. [10]. These significant differences found in transcript levels of *TPI *and *PMT *of intact plants were maintained when comparing grafted *irTPI*/*irTPI *and *irPMT*/*irPMT *plants to grafted WT/WT plants, respectively. *irTPI*/*irTPI *and *irPMT*/*irPMT *plants yield only 0.8% and 3% of *TPI *and *PMT *transcript levels of WT/WT plants, respectively (Figure 3A, B).

Leaves of *irTPI*/WT grafts (Figure 3A) failed to accumulate transcripts of *TPI *and didn't differ significantly from leaves of intact *irTPI *and grafted *irTPI*/*irTPI *plants (Fisher's PLSD test; *p *> 0.98). In addition, *TPI *transcript levels of scions of WT/*irTPI *plants (Figure 3A) didn't significantly differ from those found in WT or grafted WT/WT leaves (Fisher's PLSD test; *p *= 0.25 and *p *= 0.37, respectively). Roots of WT/*irPMT *grafts (Figure 3B) expressed similarly low levels of *PMT *transcripts when compared to the roots of *irPMT *and *irPMT*/*irPMT *grafted plants (Fisher's PLSD test; *p *= 0.92 and *p *= 0.85, respectively). However, the same low levels of *PMT *transcript (Figure 3B) were found in WT rootstocks of *irPMT*/WT plants, which didn't differ significantly from *irPMT *and *irPMT*/*irPMT *(Fisher's PLSD test; *p *= 0.94 and *p *= 0.86, respectively).

Transcript levels of *TPS *in shoot or roots of non-grafted *ovTPS *plants (Figure 3C) didn't differ significantly from those found in scions or rootstocks of grafted *ovTPS*/*ovTPS *(Fisher's PLSD test; *p *= 0.29 and *p *= 0.09, respectively). In addition, levels of *TPS *transcripts in scions of WT/*ovTPS *and rootstocks of *ovTPS*/WT (Figure 3C) resembled those of WT or WT/WT plants (Fisher's PLSD test; *p *> 0.96).

## Discussion

As previously described for other species [12,17,18], including *N. benthamiana *[19,20], here we describe a simple and highly efficient micrografting protocol for *N. attenuata *with transgenic lines. Our data support the employment of *N. attenuata *grafts that combine WT genotype either to *ov*- or *ir*-transgenic lines, this latter only when used as rootstocks, given the altered transcript accumulation of WT roots promoted by silenced shoots. The motivation to adopt this well-known grafting method for *N. attenuata *is grounded in the several layers of inter-related plant defenses that have described in *N. attenuata*. Systemic signals produced upon herbivore attack enhance the indigestibility of *N. attenuata *leaves by augmented TPI activity [8] and increase its toxicity by producing nicotine [10] and diterpene glucosides [21]. Systemic defense signals are also involved in recruiting predators to attacking herbivores by producing volatiles that betray the location of the herbivores on attacked plants [6,22], as well as in reallocation of energy to roots that are later remobilized for reproduction and thereby enhances a plant's tolerance of herbivore attack [23]. In addition, it is known that *N. attenuata *can change its flower opening time in order to recruit new pollinators that do not have larval stages that are herbivores of the plant and thereby reduces future herbivore loads [24]. Taken together, the establishment of micrografting method for *N. attenuata *will allow us to examine the systemic signals and gene function in above- versus below-ground parts of a plant in an ecological context.

Meristematic activity in the graft junction is an important determinant of attaining high grafting success rates [25]. The reason why previous attempts of cleft grafting with adult *N. attenuata *plants resulted in low success rate may be due to poor callus formation in stem tissues. The hypocotyls of germinated seeds have proved to be the most reliable explant to induce meristematic activity for tissue culture and regeneration of *N. attenuata *[26] and this tissue's particularly high meristematic activity appears also to be the reason behind the success of the of the grafting procedure described here (Figure 1C).

Other than its simplicity and high efficiency, a major advantage of this method for *N. attenuata *is that its impact on a plant's adult life is expected to be minor because the grafting takes place in an early phase of plant development. Only five to six days after grafting, completely healed and healthy grafted seedlings are obtained (Figure 1B), which do not show morphological or fitness compromises when compared to intact WT *N. attenuata *plants at later stages in development (Figure 2). Moreover, the wounding inflicted by the grafting procedure itself could potentially lead to activation of defense related traits such as augmented *TPI *expression or nicotine accumulation [27,28]. However *TPI *and *PMT *transcript accumulation levels of five-week-old WT/WT resembled those of intact WT *N. attenuata *plants (Figure 3A, B). In addition, given that the graft junction is established at the shoot-root interface, this protocol allows for the manipulation of a larger long-distance signaling system in plants, rather than only within shoots [29].

Recent molecular studies suggest the graft hybridization can occur by the exchange of genetic material among neighboring cells and across the graft junction [30-32]. However, as reported for *N. benthamiana*, micrografted roots harboring 35S-derived RNAi constructs were unable to promote silencing of its target in nonsilenced shoots [19]. To validate the micrografting method for *N. attenuata *with transgenic lines, transmission of the silencing effect in rootstocks to scions or *vice versa *should be examined. Our data are consistent with the lack, or very weak upward transmission of the silencing signal (Figure 3A). On the other hand, when *irPMT *shoots were grafted onto WT roots of one-week-old seedlings, the roots' ability to accumulate *PMT *transcripts in later developmental stages was reduced (Figure 3B), consistent with the concept of source-to-sink facilitated movement of sRNA silencing signals [32]. Regardless of the molecular mechanism underlying the spread of the silencing from shoot to root observed in *irPMT*/WT grafts, these data suggest a limitation to the use of grafts consisting of *ir *construct-derived transgenic lines scions and WT rootstocks for addressing ecological questions.

Roots are thought to play a role in the control of developmental processes of the aboveground parts of plants, such as shoot branching and flowering [12,33]. As for plant defenses, it has been shown that roots of *N. attenuata *account for both plant resistance (e.g. nicotine production, [10]) and tolerance (e.g. changes in within-plant carbon allocation, [23]) to herbivore attack. However, micrografting can further extend our understanding of a plant's below-ground interactions and molecular mechanisms and their final contribution to the whole-plant performance. For instance, the signaling underlying the JA-induced nicotine synthesis in the roots of *N. attenuata *after leaf damage can be investigated by analyzing grafted plants that have WT shoots and roots deficient in JA perception (*irCOI1*, [34]), synthesis (*irLOX3*, [35]) or activation (i.e. conversion of JA to its active JA-Ile form, [36]) (Additional file 1). In addition, ethylene is known to attenuate MeJA-induced accumulation of *PMT *transcripts as well as the production of nicotine [9]. Given the readily available ethylene-related transgenic lines (*ovETR1 *and *irACO*, [37]) of *N. attenuata *(Additional file 1), it would be interesting to determine whether ethylene biosynthesis or perception in the roots is the limiting step in the regulation of nicotine synthesis upon herbivore attack. The movement of small RNA is also important in long-distance signaling. Silenced lines of RNA-directed RNA polymerase genes (*irRdR1, irRdR2 *and *irRdR3*, [38-40]) enable us to find small RNAs that move from WT shoot to small RNA-deficient root after herbivore attack. In addition, silenced lines of Dicer-like (DCL) proteins, which will be available soon, are also useful in manipulating the role of small RNA in shoot-root signaling. Finally, scrutiny of field-grown micrografted *N. attenuata *plants displaying markedly differences in performance could lead to the identification of novel root-derived traits that account for plants' Darwinian fitness as well as serve as a complementary approach to the molecular characterization of genes [12]. Therefore we predict that the protocol reported here will be valuable for unraveling potential root-based traits that profoundly affect plant development and fitness.

## Conclusions

Micrografting combined with a collection of available stably transformed lines, especially for a non-model plant such as *N. attenuata *which has no available mutant libraries, represents a key tool to evaluate gene-function in the many developmental and physiological processes that are governed by long-distance signals. The ectopic expression of *ov *or *ir *constructs was restrained in WT/*ov, ov*/WT and WT/*ir *grafts. Micrografting thus represents an important advance towards organ-specific characterization of gene function and detection of currently unrecognized long-distance signals, particularly focusing on root physiology which determines the relationships between their below- and above-ground parts and the contribution of this root-shoot communication to whole-plant performance. Given the potential of this method in unraveling root and shoot interplay and the hitherto overlooked importance of roots, we envisage that this procedure will be commonly applied to the study of gene function in *N. attenuata*.

## Materials and methods

### Plant materials and growth

*N. attenuata *seeds are derived from an inbred collection from the DI Ranch, Utah [41]. Seeds of additional *Nicotiana *sp. were kindly supplied by Dr. Verne A. Sisson (Oxford Tobacco Research Station, Oxford, NC) and originated from collections made by Dr. T. H. Goodspeed [42]. For *TPI *(Accession number: AY426751) and *PMT *(Accession number: AF280402) silencing in *N. attenuata*, inverted repeat constructs (IR) containing either a fragment of the *TPI *gene (*irTPI*) or a consensus fragment for the two *N. attenuata*'s *PMT *genes (*irPMT*) in inverted orientation were used and characterized by Zavala et al. [15] and Steppuhn et al. [10], respectively. As previously described by these authors, these transgenic lines are not morphologically distinguishable from WT plants and show reduced resistance to herbivores. Transcript levels of *TPI *and *PMT *are found reduced to below 1% in *irTPI *and 18% in *irPMT *plants, respectively, of levels found in WT plants. Consistent with the transcript analysis, no TPI activity was detected in leaf tissues of *irTPI *plants while levels of nicotine were reduced to 15% in leaves of *irPMT *plants [16]. *TPS10*-overexpressing *N. attenuata *plants (*ovTPS*) harbor a sense sequence based on the *TPS10 *gene of maize (Accession number: AY928078), driven by CaMV 35S promoter [7]. The maize *TPS10 *gene encodes a terpene synthase involved in the herbivory-induced production of (*E*)-α-bergamotene and (*E*)-β-farnesene which are the major components of the volatile blend emitted by attacked plants and function as attractants of herbivore's natural enemies [43]. As for the *ir *lines included in this study, no morphological or developmental differences between WT and *ovTPS *are observed [7]. All seeds were sterilized and treated with 0.1 mM gibberellic acid in 1:50 smoke-distilled water solution for 1 h [26]. Petri dishes containing 40 mL of Gamborg's B5 media with minimal organics containing 0.8% (w/v) plant agar (Duchefa) were used for germination, manipulation and growth of grafted seedlings. The plates were maintained in growth chambers (Percival, Perry Iowa, USA) at 26 ± 2°C, under 16/8 h of light/dark regime and plants were further transferred to the glasshouse under same conditions when necessary. Grafts were performed in sterile bench under an Olympus SZ51 stereomicroscope. Seedling pictures were obtained using a stereomicroscope equipped with a digital CCD camera (SteREO Discovery.V8, 14 Carl Zeiss Microimaging) and processed with AxioVision LE software (Carl Zeiss 15 Microimaging). Growth parameters were measured weekly (n = 10). Flowers were counted weekly from the 6^th ^until the 8^th ^week of development and the total number of produced flowers was compared. For seed production, mature capsules were collected from plants (n = 5) over 5 days starting from the first mature capsule harvested.

### RNA isolation and real time quantitative PCR (qPCR)

RNA was extracted from shoots of twenty-day-old or from leaves of five-week-old plants using Tri Reagent [44]. For root RNA extraction, the Tri protocol was modified: 300 mg of ground frozen root material was used and one extra round of centrifugation (12000 g, 15 min) was adopted before addition of chloroform. Extracted RNA was checked on agarose gel and quantified with a NanoDrop ND-1000 spectrophotometer (NanoDrop Technologies, Wilmington, DE, USA). Synthesis of cDNA from 0.5 μg of RNA per sample and qPCR analyses were conducted as in Wu et al. [45] using a Mx3005P qPCR system (Stratagene, Santa Clara, CA, USA, http://www.stratagene.com) and qPCR Core Kit for SYBR^® ^Green I (Eurogentec, Seraing, Belgium, http://www.eurogentec.com). Transcript levels were quantified relative to *N. attenuata *elongation factor 1A (NaeEF1A) and primers were designed according to Steppuhn et al. [10] and Zavala et al. [15]. All reactions were performed with at least 5 biological replicates.

### Statistical analysis

After verifying data for *t*- or normal distribution, Welch's *t*-test or Kruskall-Wallis test, followed by Fisher's PLSD as a *post-hoc *test, were performed using R-2.11.1 http://www.R-project.org or StatView5 softwares (SAS Institute, Cary, NC, USA).

## Competing interests

The authors declare that they have no competing interests.

## Authors' contributions

VF carried out the lab work, HG helped with the *irTPI *and *irPMT *grafts, ITB and SK conceived the project and oversaw the research. All authors wrote, read and approved the final manuscript.

## Supplementary Material

Additional file 1 **Table 1: List of stably transformed lines of *Nicotiana attenuata***. Lines harbor sense (*ov*), antisense (*as*) or inverted repeats (*ir*) constructs and were created in two different accessions of natural population (Arizona, Az or Utah, Ut) [46-74]. Click here for file
