# Supplementary material for: A simple and efficient micrografting method for stably transformed Nicotiana attenuata plants to examine shoot-root signaling
Source: Plant Methods. 2011 Oct 20;7:34. doi: 10.1186/1746-4811-7-34 (PMC3207920; doi:10.1186/1746-4811-7-34)
Supplement: Additional file 1 — Table 1: List of stably transformed lines of Nicotiana attenuata. Lines harbor sense (ov), antisense (as) or inverted repeats (ir) constructs and were created in two different accessions of natural population (Arizona, Az or Utah, Ut) [46-74]. [file 1746-4811-7-34-S1.PDF]

Table 1: List of stably transformed lines of *Nicotiana attenuata.*

Lines harbor sense (*ov*), antisense (*as*) or inverted repeats (*ir*) constructs and were created in two different accessions of natural population (Arizona, Ar or Utah, Ut)

Line	Accession	Vector/Cross	Selectable marker	Gene(s)	Publication
Empty Vector (EV)	Ar and Ut	pRESC2NC	hygromycin	empty vector	[15]
Empty Vector (EV)	Ut	pNATNC	nourseothricin	empty vector	[15]
Empty Vector (EV)	Ut	pCAMBIA1300	hygromycin	empty vector	[46]
Empty Vector (EV)	Ut	pSOL3NC	hygromycin	empty vector	[47]
asLOX3	Ut	pNATLOX	nourseothricin	lipoxygenase 3	[48]
ovHPTII	Ut	pCAMBIA1300	hygromycin	hygromicin phosphotransferase II	[49]
ovGUS	Ut	pCAMBIA1301	GUS	β-glucuronidase A	[49]
asAOS	Ut	pNATAOS2	nourseothricin	allene oxide synthase	[50]
asHPL	Ut	pNATHPL1	nourseothricin	hydroperoxide lyase	[50]
asTPI	Ut	pNATPI1	nourseothricin	trypsin proteinase inhibitor	[15]
ovTPI	Ar	pRESC2PIA2	hygromycin	trypsin proteinase inhibitor	[15]
asPMT	Ut	pCAMPMT1	hygromycin	putrescine *N*-methyl transferase	[10]
irPMT	Ut	pRESC5PMT	hygromycin	putrescine *N*-methyl transferase	[10]
asGAL83	Ut	pNATGAL83	nourseothricin	β-subunit of SNF1-related kinase	[23]
asGLP	Ut	pRESC2GER	hygromycin	germin-like protein	[51]
asTD	Ut	pNATTD1	nourseothricin	threonine deaminase	[52]
TDpromoter::GUS	Ut	pCAMBIA1301	hygromycin	threonine deaminase promoter::GUS	[46]
irRAFL	Ut	pRESC5RALF	hygromycin	rapid alkalinization factor	[53]
irCOI1	Ut	pSOL3COI1	hygromycin	coronatine insensitive 1	[34]
irACO	Ut	pRESC5ACO1	hygromycin	ACC oxidase	[37]
sETR1	Ut	pRESC2ETR1	hygromycin	ethylene receptor 1	[37]
irJAR4	Ut	pRESC5JAR4	hygromycin	jasmonate resistant 4	[54]
irJAR6	Ut	pRESC5JAR6	hygromycin	jasmonate resistant 6	[54]
irPMT	Ut	pRESC5PMT	hygromycin	putrescine *N*-methyl transferase	[16]
irTPI	Ut	pSOL3PI	hygromycin	trypsin proteinase inhibitor	[16]
irPMT/irTPI	Ut	pRESC5PMT::pSOL3PI	hygromycin	putrescine *N*-methyl transferase::trypsin proteinase inhibitor	[16]
irPMT/irTPI	Ut	pRESC5PMT::pSOL4PI	nourseothricin	putrescine *N*-methyl transferase::trypsin proteinase inhibitor	[16]
irNPR1	Ut	pRESC5NPR1	hygromycin	non-expressor of PR-1	[55]
irRdR1	Ut	pRESC5RdR1	hygromycin	RNA-directed RNA polymerase 1	[38]
irSYS	Ut	pRESC5SYS2	hygromycin	Systemin (preproTobHypSys)	[56]
ovSYS	Ut	pRESC2SYS2	hygromycin	Systemin (preproTobHypSys)	[56]
irJAR4/irJAR6	Ut	*irJAR4* x *irJAR6*	hygromycin	jasmonate resistant 4 and jasmonate resistant 6	[36]
irPMT	Az	pRESC5PMT	hygromycin	putrescine *N*-methyl transferase	[57]
ovTPI	Az	pRESC2PIA2	hygromycin	trypsin proteinase inhibitor	[57]
ovWRKY3	Ut	pRESC2WRKY3	hygromycin	WRKY transcription factor	[58]
ovWRKY6	Ut	pRESC2WRKY6	hygromycin	WRKY transcription factor	[58]
irWRKY3	Ut	pSOL3WRKY3	hygromycin	WRKY transcription factor	[58]
irWRKY6	Ut	pSOL3WRKY6	hygromycin	WRKY transcription factor	[58]
irWRKY3/irWRKY6	Ut	*irWRKY3* x *irWRKY6*	hygromycin	WRKY transcription factor	[58]
irPR-1	Ut	pRESC5PR1	hygromycin	pathogenesis-related protein 1	[59]
irThionin	Ut	pRESC5Thionin	hygromycin	thionin (pathogenesis-related protein 13)	[59]
irDefensin	Ut	pRESC5Defensin	hygromycin	defensin (pathogenesis-related protein 12)	[60]
irRdR3	Ut	pRESC5RdR3	hygromycin	RNA-directed RNA polymerase 3 (RdR6 homolog)	[40]
irRdR2	Ut	pRESC5RdR2	hygromycin	RNA-directed RNA polymerase 2	[39]
asRUB	Ut	pRESC2RUB	hygromycin	ribulose-1,5-bisphosphate carboxylase/oxygenase	[61]
irRCA	Ut	pRESC5RCA	hygromycin	RuBPCase activase (RCA)	[61]
irCHAL	Ut	pRESC5CHAL	hygromycin	chalcone synthase	[47]
irCHAL	Ut	pRESC1CHAL	nourseothricin	chalcone synthase	[47]
irPMT/irCHAL	Ut	*irPMT* x *irCHAL*	nourseothricin	putrescine *N*-methyl transferase and chalcone synthase	[47]
irSIPK	Ut	pRESC5SIPK	hygromycin	salicylic acid-induced protein kinase	[62]
irWIKP	Ut	pRESC5WIPK	hygromycin	wound-induced protein kinase	[62]
asLOX3/asHPL	Ut	*asLOX3* x *asHPL*	nourseothricin	lipoxygenase 3 and hydroperoxide lyase	[62]
irPME	Ut	pRESC5PME1	hygromycin	pectin methylesterases	[63]
irSYS	Az	pRESC5sys2	hygromycin	Systemin (preproTobHypSys)	[64]
irαDOXS	Ut	pRESC5αDOX1	hygromycin	α-dioxygenase 1	[65]
irαDOXM	Ut	pRESC5αDOX1	hygromycin	α-dioxygenase 1	[65]
irLOX2	Ut	pSOL3LOX2	hygromycin	lipoxygenase 2	[66]
irLOX3	Ut	pRESC5LOX3	hygromycin	lipoxygenase 3	[66]
irLOX2/irLOX3	Ut	*irLOX2* x *irLOX3*	hygromycin	lipoxygenase2 and lipoxygenase3	[35]
irSIPK/irWIPK	Ut	*irSIPK* x *irWIPK*	hygromycin	salicylic acid-induced protein kinase and wound-induced protein kinase	[22]
sETR1/asLOX3	Ut	*sETR1* x *asLOX3*	hygromycin	lipoxygenase 3 and ethylene receptor 1	[67]
irMYB8	Ut	pSOL8MYB8	hygromycin	MYB transcription factor	[68]
irGGPPS	Ut	pRESC5GGPPS	hygromycin	geranylgeranyl diphosphate	[21]
irNOA1	Ut	pSOL8NOA1	hygromycin	nitric oxide-associated protein 1	[69]
irMYB3	Ut	pSOL8MYB3	hygromycin	R2R3-MYB transcription factor	[70]
irGLA1	Ut	pSOL8GLA1	hygromycin	glycerolipase A1	[71]
irHPL	Ut	pSOL3HPL	hygromycin	hydroperoxide lyase	[72]
ovJMT	Ut	pRESC2JMT	hygromycin	JA O-methyltransferase	[73]
ovJMT/irMJE	Ut	pRESC2JMT::pSOL8MJE	hygromycin	JA O-methyltransferase::methyl jasmonate esterase	[73]
irLecRK	Ut	pSOL8LECRK	hygromycin	lectin receptor-like kinase	[74]
ovNahG	Ut	pSOL1NAHG1	hygromycin	salycilate hydroxylase	[74]
irLecRK/ovNahG	Ut	*irLECRK x ovNahG*	hygromycin	lectin receptor-like kinase and salycilate hydroxylase	[74]
